# Prognostic and immune infiltration significance of ARID1A in TCGA molecular subtypes of gastric adenocarcinoma

**DOI:** 10.1002/cam4.6294

**Published:** 2023-06-27

**Authors:** Zhenkun Zhang, Qiujing Li, Shanshan Sun, Jing Ye, Zhe Li, Zhengguo Cui, Qian Liu, Yujie Zhang, Sili Xiong, Shukun Zhang

**Affiliations:** ^1^ Weihai Municipal Hospital Shandong University Weihai China; ^2^ Department of Oncology Shouguang People's Hospital Weifang China; ^3^ Department of Pathology, Weihai Municipal Hospital Shandong University Weihai China; ^4^ Department of Oncology, Weihai Municipal Hospital Shandong University Weihai China; ^5^ Binzhou Medical University Yantai China; ^6^ Weifang Medical College Weifang China; ^7^ Department of Environmental Health University of Fukui School of Medical Sciences Fukui Japan

**Keywords:** ARID1A, gastric adenocarcinoma, immune infiltration, prognosis, TCGA subtype

## Abstract

**Background:**

AT‐rich interaction domain 1A (ARID1A) is an essential subunit of the switch/sucrose non‐fermentable chromatin remodeling complex and is considered to be a tumor suppressor. The Cancer Genome Atlas (TCGA) molecular classification has deepened our understanding of gastric cancer at the molecular level. This study explored the significance of ARID1A expression in TCGA subtypes of gastric adenocarcinoma.

**Methods:**

We collected 1248 postoperative patients with gastric adenocarcinoma, constructed tissue microarrays, performed immunohistochemistry for ARID1A, and obtained correlations between ARID1A and clinicopathological variables. We then carried out the prognostic analysis of ARID1A in TCGA subtypes. Finally, we screened patients by random sampling and propensity score matching method and performed multiplex immunofluorescence to explore the effects of ARID1A on CD4, CD8, and PD‐L1 expression in TCGA subtypes.

**Results:**

Seven variables independently associated with ARID1A were screened out: mismatch repair proteins, PD‐L1, T stage, differentiation status, p53, E‐cadherin, and EBER. The independent prognostic variables in the genomically stable (GS) subtype were N stage, M stage, T stage, chemotherapy, size, and ARID1A. PD‐L1 expression was higher in the ARID1A negative group than in the ARID1A positive group in all TCGA subgroups. CD4 showed higher expression in the ARID1A negative group in most subtypes, while CD8 did not show the difference in most subtypes. When ARID1A was negative, PD‐L1 expression was positively correlated with CD4/CD8 expression; while when ARID1A was positive, this correlation disappeared.

**Conclusions:**

The negative expression of ARID1A occurred more frequently in the Epstein–Barr virus and microsatellite instability subtypes and was an independent adverse prognostic factor in the GS subtype. In the TCGA subtypes, ARID1A negative expression caused increased CD4 and PD‐L1 expression, whereas CD8 expression appeared independent of ARID1A. The expression of CD4/CD8 induced by ARID1A negativity was accompanied by an increase in PD‐L1 expression.

## 1 INTRODUCTION

According to the latest global cancer statistics report, the incidence of gastric cancer ranks fifth, and gastric cancer‐related mortality ranks fourth worldwide, seriously endangering human health.^1^ Despite obvious advances in diagnostic techniques and treatments in recent years, the overall prognosis of gastric cancer remains unsatisfactory. With the development of molecular biology techniques, The Cancer Genome Atlas (TCGA) proposed the concept of molecular classification, which was classified into four subtypes in gastric cancer: Epstein–Barr virus (EBV) positive subtype, microsatellite instability (MSI) subtype, genomically stable (GS) subtype, and chromosomal instability (CIN) subtype.^2^ The TCGA molecular classification not only greatly expands our knowledge on the heterogeneity and molecular complexity of gastric cancer but also shows important prognostic and therapeutic significance. Considering the high‐throughput techniques used in TCGA classification are very complex and expensive to apply on a large scale in clinical practice, several studies have proposed the immunohistochemistry (IHC) and EBV‐encoded RNA in situ hybridization (EBER‐ISH) techniques as an alternative for routine application in pathology laboratories.^3^, ^4^

The switch/sucrose non‐fermentable (SWI/SNF) chromatin remodeling complex dynamically alters the structure of chromatin, allowing highly condensed chromatin to expose more accessible sites for DNA binding factors, and further controlling gene expression.^5^ AT‐rich interaction domain 1A (ARID1A) is a core component of the SWI/SNF complex, which plays an essential role in binding the SWI/SNF complex to DNA and is involved in regulating many critical cellular processes, such as cell proliferation, cell differentiation, and DNA repair.^6^ The ARID1A protein is encoded by the eponymous *ARID1A* gene, which is most frequently mutated among genes encoding for the SWI/SNF complex subunits,^5^ and its mutation frequency is ranked only second to that of *TP53* in gastric cancer.^2^, ^7^ In recent years, gastric adenocarcinoma with ARID1A abnormalities has received increasing attention as a distinct tumor entity.

Currently, there are many studies on ARID1A in gastric adenocarcinoma, while the significance of ARID1A in the TCGA molecular subtypes of gastric adenocarcinoma has not been studied in depth. Herein, we explored the prognostic and immune infiltration significance of ARID1A negative expression in TCGA subtypes in 1248 patients with gastric adenocarcinoma.

## 2 MATERIALS AND METHODS

### 2.1 Patients collection and tissue microarrays construction

We collected 1347 patients with gastric adenocarcinoma who received initial surgical treatment at Weihai Municipal Hospital between January 2014 and December 2020. Subsequently, we excluded patients who received antitumor therapy before surgery (e.g., chemotherapy, radiotherapy, immunotherapy, molecular targeted therapy, etc.). The tissue microarrays (TMA) were constructed as follows. First, two pathologists carefully reviewed the hematoxylin and eosin (H&E)‐stained slides of the screened patients and marked representative tumor areas. Second, we took out one tissue core (2 mm in diameter) from each corresponding formalin‐fixed and paraffin‐embedded (FFPE) donor block using a manual tissue sampling gun (jlm‐5133, Guangdong, China) and transferred the donor tissue cores to the recipient paraffin block (ZSGB‐BIO, 60 holes, 2 mm in diameter). Finally, we fused the donor cores and the recipient block into TMAs by heating slowly to 65°C. After excluding the spots that did not contain tumor tissue and those that detached from the TMA slides, 1248 patients were included in the study.

We obtained the following clinical pathological information by reviewing the electronic medical record (EMR) systems and pathological records: age, sex, tumor site, tumor size, differentiation status, WHO histological classification, Lauren classification, vascular invasion (VI), perineural invasion (PNI), invasion depth, lymph node metastasis, and distant organ metastasis. We restaged the enrolled patients according to the latest AJCC TNM staging system (8th edition, 2019). Overall survival (OS) times was defined as the time from the date of diagnosis to the date of death or last follow‐up, which were obtained by EMR system or telephone follow‐up.

**FIGURE 1:**
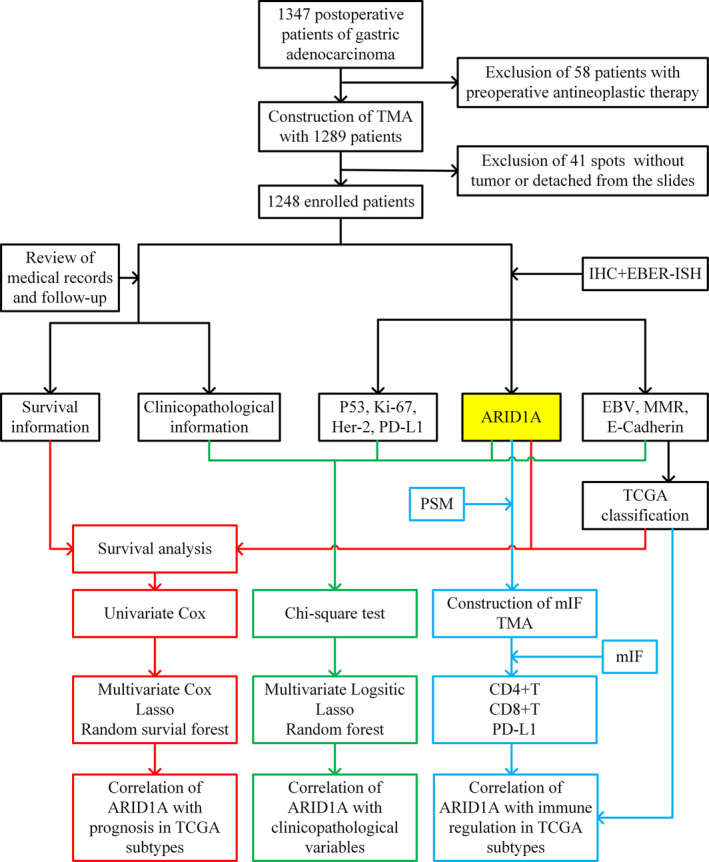
Study design and workflow of the present study. EBER‐ISH, EBV‐encoded RNA in situ hybridization; EBV, Epstein–Barr virus; Her‐2, human epidermal growth factor receptor 2; IHC, immunohistochemistry; mIF, multiplex immunofluorescence; MMR, mismatch repair; PD‐L1, programmed cell death ligand‐1; PSM, propensity score matching; TCGA, The Cancer Genome Atlas; TMA, tissue microarrays.

This study was approved by the Ethics Review Board of Weihai Municipal Hospital (permission code: 2021053), which considered that the written informed consent from patients was unnecessary due to this study was retrospective and was conducted anonymously. The design and workflow of this study are shown in Figure 1.

### 2.2 Immunohistochemistry and in situ hybridization

We obtained sections of 2 μm thickness from TMA blocks and then implemented IHC staining for ARID1A, E‐cadherin, p53, PD‐L1, Ki‐67, HER‐2, MSH2, MSH6, MLH1, and PSM2 using an automated immunostaining machine (Benchmark ULTRA, Ventana) following the manufacturer's procedure. The primary antibodies' information were as follows, ARID1A (EPR13501, Rabbit, 1:1000, Abcam), E‐cadherin (EP6, Rabbit, Prediluted, Origene), MSH2 (RED2, Rabbit, Prediluted, Origene), MSH6 (EP49, Rabbit, Prediluted, Origene), MLH1 (ES05, Rabbit, Prediluted, Origene), PMS2 (EP51, Rabbit, Prediluted, Origene), p53 (DO‐7, Mouse, Prediluted, Origene), PD‐L1 (SP263, Rabbit, Prediluted, Roche), Ki‐67 (MyM1‐Ki67, Mouse, Prediluted, Anbiping), and Her‐2 (4B5, Rabbit, Prediluted, Roche). If the immunohistochemical staining of Her‐2 is 2+, further fluorescence in situ hybridization (FISH) is required. We detected EBV infection by the EBER‐ISH method using the EBER assay kits (ZSGB‐BIO, ISH‐7001).

### 2.3 Assessment criteria for immunohistochemical staining

ARID1A was scored according to staining intensity and staining proportion of tumor cells, respectively. Staining intensity was scored as 0 (absence of any nuclear staining), 1 (faint nuclear staining), and 2 (intense nuclear staining); Staining proportion was scored as 0 (0%), 1 (1%–10%), and 2 (11%–100%). The above two scores were multiplied to obtain a final score, ranging from 0 to 4; 0 or 1 was defined as ARID1A negative, and ≥2 was defined as ARID1A positive.^8^ For heterogeneous expression, the score of each component was multiplied by the proportion and then summed to obtain the final score. MMR proteins (including MLH1, PMS2, MSH2, and MSH6) were classified as present (definite nuclear staining) and lost (complete absence of nuclear staining). Any MMR proteins lost were defined as MMR deficient (dMMR), and all MMR proteins present were defined as MMR proficient (pMMR). Complete absence or apparent reduction (>30%) of membranous staining for E‐cadherin was evaluated as absent, irrespective of cytoplasmic staining.^3^ The evaluation criteria for p53 were as follows: nuclear staining of inhomogeneous variable intensity was defined as wild‐type, and either complete absence of nuclear staining in all tumor cells or strong diffuse nuclear staining in more than 90% of tumor cells was defined as mutant‐type.^9^ The criteria for IHC evaluation of HER2, Ki‐67, and PD‐L1 are listed in Table S1.

### 2.4 TCGA molecular classification

We performed an analogous TCGA molecular classification according to the staining results of EBER, MMR, and E‐cadherin. First, we selected EBER‐positive cases as EBV subtype; Next, we classified the dMMR cases into MSI subtype based on the MMR result; Then, we classified cases with E‐cadherin negative expression as GS subtype; Finally, the remaining cases were classified as CIN subtype.^3^, ^4^

### 2.5 Multiplex immunofluorescence (mIF)

We randomly screened half of the ARID1A negative patients, followed by screening ARID1A positive patients using the propensity score matching (PSM) method in a 1:1 ratio. We then marked interest regions in tumor center (TC) and invasive margin (IM) on representative H&E‐stained slides. Finally, we constructed TMAs using the same method described above for subsequent mIF analysis.

The mIF for CD4, CD8, PD‐L1, CK‐pan, and DAPI was performed at Tissuegnostics Asia Pacific Limited (Beijing, China) using a five‐color kit from Tissuegnostics (commercial number: TGFP550) following the manufacturer's protocol. Immunofluorescence images were acquired using the Tissue FAXS Cytometry platform of Tissuegnostics and were then high‐throughput quantitatively analyzed with Strata Quest software (version 7.0.1). The brief protocol was as follows: First, the fluorescence was detected by fluorescence microscope at different wavelengths in five channels respectively, dark blue for DAPI (TG470SN), skyblue for CK pan (TG430N), green for CD4 (TG520N), yellow for CD8 (TG570N), and red for PD‐L1 (TG650N). Next, the DAPI fluorescence staining channel was used for nucleus identification, and the parameters were adjusted by the “forward‐reverse tracking tools” according to the results of quantitative analysis to obtain the cell number in sight (n/sight). Then, the image segmentation of the nucleus and cytoplasm was performed, and optimal thresholds for the positivity of each marker were determined based on fluorescence signal intensity. Finally, each marker's positive cells (n/sight) were calculated according to the fluorescence signal around the nucleus.

### 2.6 Statistical analysis

All statistical analyses and statistical graphics were performed with R software (version 4.1.2). The chi‐square test was used to analyze the correlation between categorical variables, including the correlation between ARID1A and clinicopathological features, as well as the correlation between ARID1A and TCGA molecular classification. Variables with *p* < 0.05 were then screened out for the subsequent multivariate logistic regression analysis (screening variables with the stepwise method) and least absolute shrinkage and selection operator (LASSO) regression analysis, respectively. Then, a random forest model was used to sort the variables filtered out by logistic regression and LASSO according to the importance, measured by increase in node purity (IncNodePurity), with higher values indicating more significant importance.^10^ The Kaplan–Meier method was used for survival analysis and plotting of survival curves, comparing the differences in survival curves by the log‐rank test. The univariate Cox regression analysis was performed to preliminary screen variables associated with prognosis. Variables with *p* < 0.05 were then screened out for the subsequent multivariate Cox regression analysis and LASSO regression analysis, respectively. Then, a random survival forest model was used to sort the variables according to the variable importance (VIPM), with higher values indicating stronger predictive ability.^11^ The PSM method (method = “nearest”, caliper = 0.02) was used to screen ARID1A positive patients in a 1:1 ratio based on baseline characteristics of ARID1A negative patients for subsequent mIF. The independent Student's *t*‐test (data meet normal distribution and variance homogeneity) or Wilcoxon rank‐sum test was used to perform statistical analysis for continuous data in two groups. Spearman's rank test was used to analyze the correlation between immune markers (CD4, CD8, and PD‐L1). Two‐sided tests were adopted, and the differences were considered statistically significant when *p* < 0.05.

The R packages used in this study were listed as follows: ggplot2 (3.3.6), rcompanion (2.4.18), epiDisplay (3.5.0.1), MASS (7.3–54), forestmodel (0.6.2), glmnet (4.1–3), randomForest (4.7–1.1), survival (3.2–13), survminer (0.4.9), randomForestSRC (3.1.1), pheatmap (1.0.12), and MatchIt (4.3.1).

## 3 RESULTS

### 3.1 Clinicopathological information of the patients

**FIGURE 2:**
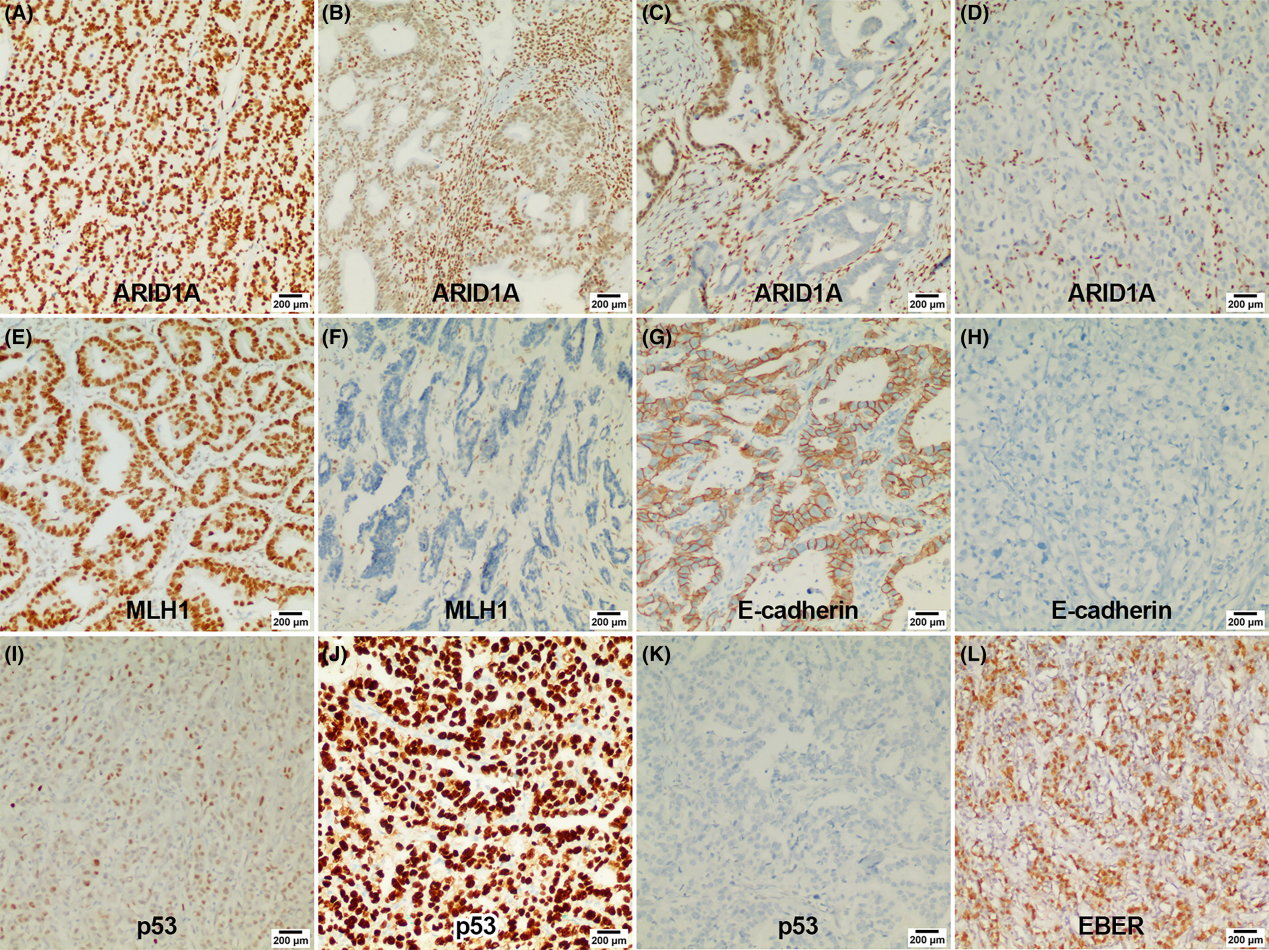
Representative immunohistochemistry images of important markers. (A) Diffuse strong expression of ARID1A; (B) reduced expression of ARID1A; (C) heterogeneous expression of ARID1A; (D) complete loss expression of ARID1A; (E) positive expression of MLH1; (F) negative expression of MLH1; (G) positive expression of E‐cadherin; (H) negative expression of E‐cadherin; (I) wild‐type expression of p53 (nuclear staining of variable intensity); (J) mutant‐type expression of p53 (diffuse and uniform strong nuclear staining); (K) mutant‐type expression of p53 (complete absence of nuclear staining); (L) positive expression of EBV. EBER, EBV‐encoded RNA; EBER‐ISH, EBV‐encoded RNA in situ hybridization.

A total of 1248 patients were enrolled in this study with a median age of 64 years (range: 28.0–88.0), nearly 3/4 (929, 74.44%) were male, and most of the lesions were located in the antrum (759, 60.82%). The distribution of AJCC TNM stage I, II, III, and IV in the cohort was 310 (24.84%), 299 (23.96%), 576 (46.15%), and 63 (5.05%), respectively. The main IHC findings were as follows: ARID1A negative in 275 patients (22.04%), EBER positive in 71 patients (5.69%), dMMR in 184 patients (14.74%), E‐cadherin absent in 418 patients (33.49%), Her‐2 positive in 79 patients (6.33%), and PD‐L1 positive in 87 patients (6.97%). The detailed clinicopathological information is shown in the “Total” column of Table 1. Typical IHC and EBER images are shown in Figure 2.

**TABLE 1:** Univariate correlation analysis of ARID1A with clinicopathological variables.

		ARID1A		
Variables	Total	Negative	Positive	*p*‐Value
Age
1248	275 (22.04)	973 (77.96)	0.458
<60	372 (29.81)	77 (28)		295 (30.32)
≥60	876 (70.19)	198 (72)		678 (69.68)
Sex				0.094
Female	319 (25.56)	81 (29.45)	238 (24.46)	
Male	929 (74.44)	194 (70.55)	735 (75.54)	
Site				0.141
Antrum	759 (60.82)	156 (56.73)	603 (61.97)	
Body	357 (28.61)	82 (29.82)	275 (28.26)	
Cardia	132 (10.58)	37 (13.45)	95 (9.76)	
Size				**0.003**
<4 cm	624 (50)	116 (42.18)	508 (52.21)	
≥4 cm	624 (50)	159 (57.82)	465 (47.79)	
T‐stage				**<0.001**
T1	227 (18.19)	23 (8.36)	204 (20.97)	
T2	185 (14.82)	47 (17.09)	138 (14.18)	
T3	204 (16.35)	41 (14.91)	163 (16.75)	
T4	632 (50.64)	164 (59.64)	468 (48.1)	
N‐stage				0.094
N0	484 (38.78)	101 (36.73)	383 (39.36)	
N1	210 (16.83)	53 (19.27)	157 (16.14)	
N2	218 (17.47)	58 (21.09)	160 (16.44)	
N3	336 (26.92)	63 (22.91)	273 (28.06)	
M‐stage				**0.026**
M0	1185 (94.95)	254 (92.36)	931 (95.68)	
M1	63 (5.05)	21 (7.64)	42 (4.32)	
TNM‐stage				**0.006**
I	310 (24.84)	51 (18.55)	259 (26.62)	
II	299 (23.96)	76 (27.64)	223 (22.92)	
III	576 (46.15)	127 (46.18)	449 (46.15)	
IV	63 (5.05)	21 (7.64)	42 (4.32)	
Differentiation				**<0.001**
Moderate	142 (11.38)	27 (9.82)	115 (11.82)	
Poor	699 (56.01)	189 (68.73)	510 (52.42)	
Well	407 (32.61)	59 (21.45)	348 (35.77)	
WHO				0.659
Tubular	642 (51.44)	141 (51.27)	501 (51.49)	
Papillary	49 (3.93)	9 (3.27)	40 (4.11)	
Poorly cohesive	498 (39.9)	115 (41.82)	383 (39.36)	
Mucinous	59 (4.73)	10 (3.64)	49 (5.04)	
Lauren				**<0.001**
Diffuse	666 (53.37)	170 (61.82)	496 (50.98)	
Intestinal	418 (33.49)	66 (24)	352 (36.18)	
Mixed	164 (13.14)	39 (14.18)	125 (12.85)	
VI				**0.004**
No	650 (52.08)	122 (44.36)	528 (54.27)	
Yes	598 (47.92)	153 (55.64)	445 (45.73)	
PNI				0.861
No	907 (72.68)	201 (73.09)	706 (72.56)	
Yes	341 (27.32)	74 (26.91)	267 (27.44)	
Her‐2				**0.018**
Negative	1169 (93.67)	266 (96.73)	903 (92.81)	
Positive	79 (6.33)	9 (3.27)	70 (7.19)	
Ki‐67				0.214
High	1021 (81.81)	232 (84.36)	789 (81.09)	
Low	227 (18.19)	43 (15.64)	184 (18.91)	
PD‐L1				**<0.001**
Negative	1161 (93.03)	227 (82.55)	934 (95.99)	
Positive	87 (6.97)	48 (17.45)	39 (4.01)	
EBER				**<0.001**
Negative	1177 (94.31)	248 (90.18)	929 (95.48)	
Positive	71 (5.69)	27 (9.82)	44 (4.52)	
MMR				**<0.001**
dMMR	184 (14.74)	95 (34.55)	89 (9.15)	
pMMR	1064 (85.26)	180 (65.45)	884 (90.85)	
p53				**<0.001**
Mutation	527 (42.23)	71 (25.82)	456 (46.87)	
Wild	721 (57.77)	204 (74.18)	517 (53.13)	
E‐cadherin				**<0.001**
Negative	418 (33.49)	128 (46.55)	290 (29.8)	
Positive	830 (66.51)	147 (53.45)	683 (70.2)	

Variables with *p* < 0.05 were shown in bold value.

Abbreviations: dMMR, mismatch repair deficient; EBER, EBV‐encoded RNA; Her‐2, human epidermal growth factor receptor 2; MMR, mismatch repair; PD‐L1, programmed cell death ligand‐1; pMMR, mismatch repair proficient; PNI, perineural invasion; VI, vascular invasion.

**FIGURE 3:**
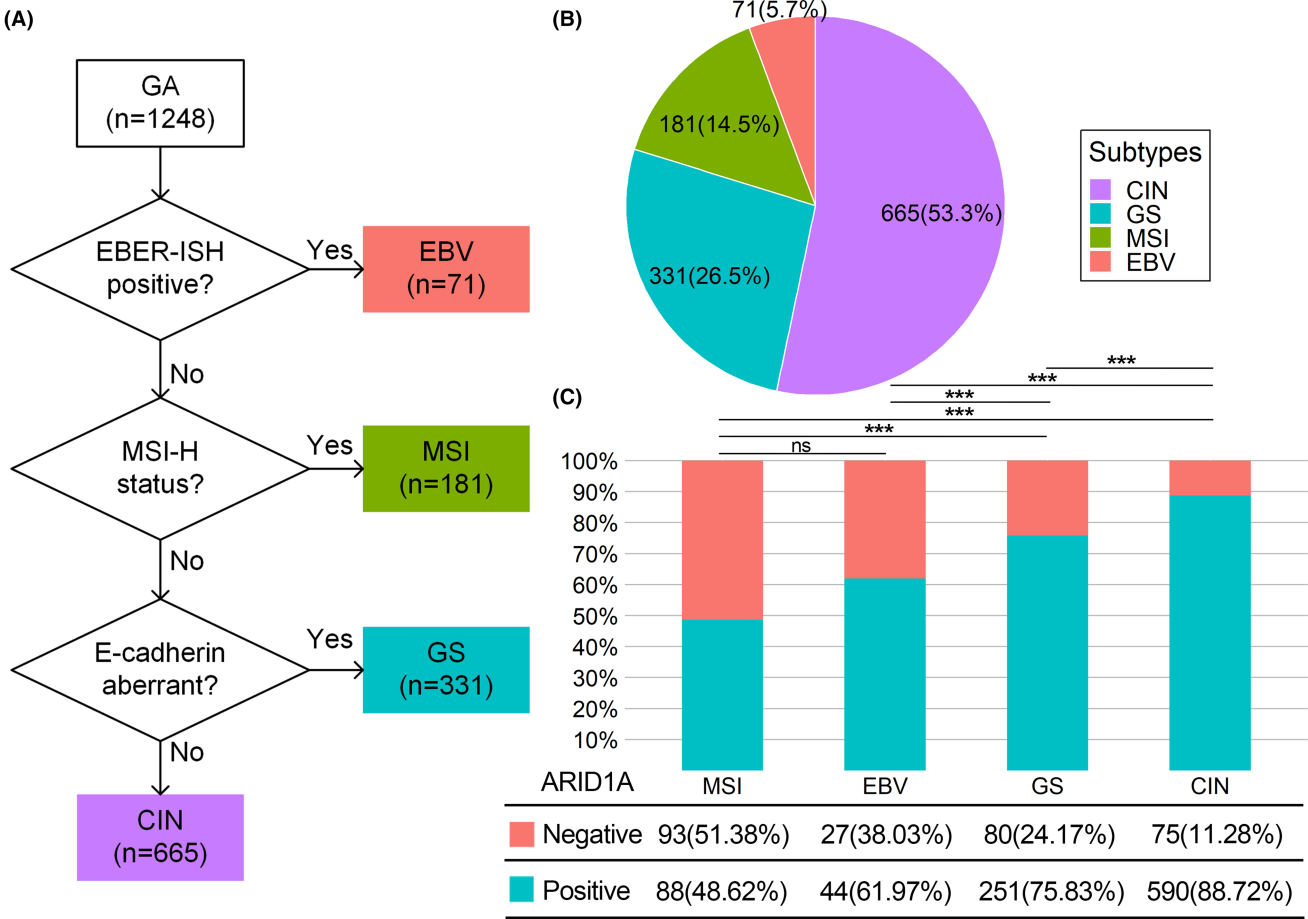
(A) Flowchart of TCGA molecular classification based on IHC and EBER‐ISH. (B) Distribution of TCGA subtypes. (C) ARID1A expression in TCGA subtypes. CIN, chromosomal instability; EBV, Epstein–Barr virus; GS, genomically stable; MSI, microsatellite instability; ns, no significance. ***: *p* < 0.001.

The distribution of EBV, MSI, GS, and CIN subtypes in the entire cohort was 71 (5.69%), 181 (14.5%), 331 (26.52%), and 665 (53.29%), respectively (Figure 3A,B). Among the TCGA subtypes, the MSI subtype showed the highest proportion of ARID1A negative (51.38%), followed by the EBV subtype (38.03%); however, there was no statistical difference between these two subtypes (Figure 3C). The specific statistical results of pairwise comparison among subtypes are shown in Table S2.

### 3.2 Correlation between ARID1A expression and clinicopathologic variables

Univariate correlation analysis between ARID1A expression and clinicopathological variables identified 13 variables with *p* < 0.05 as follows: Size, T stage, M stage, TNM stage, differentiation status, Lauren classification, VI, Her‐2, PD‐L1, EBER, MMR, p53, and E‐cadherin (Table 1).

**FIGURE 4:**
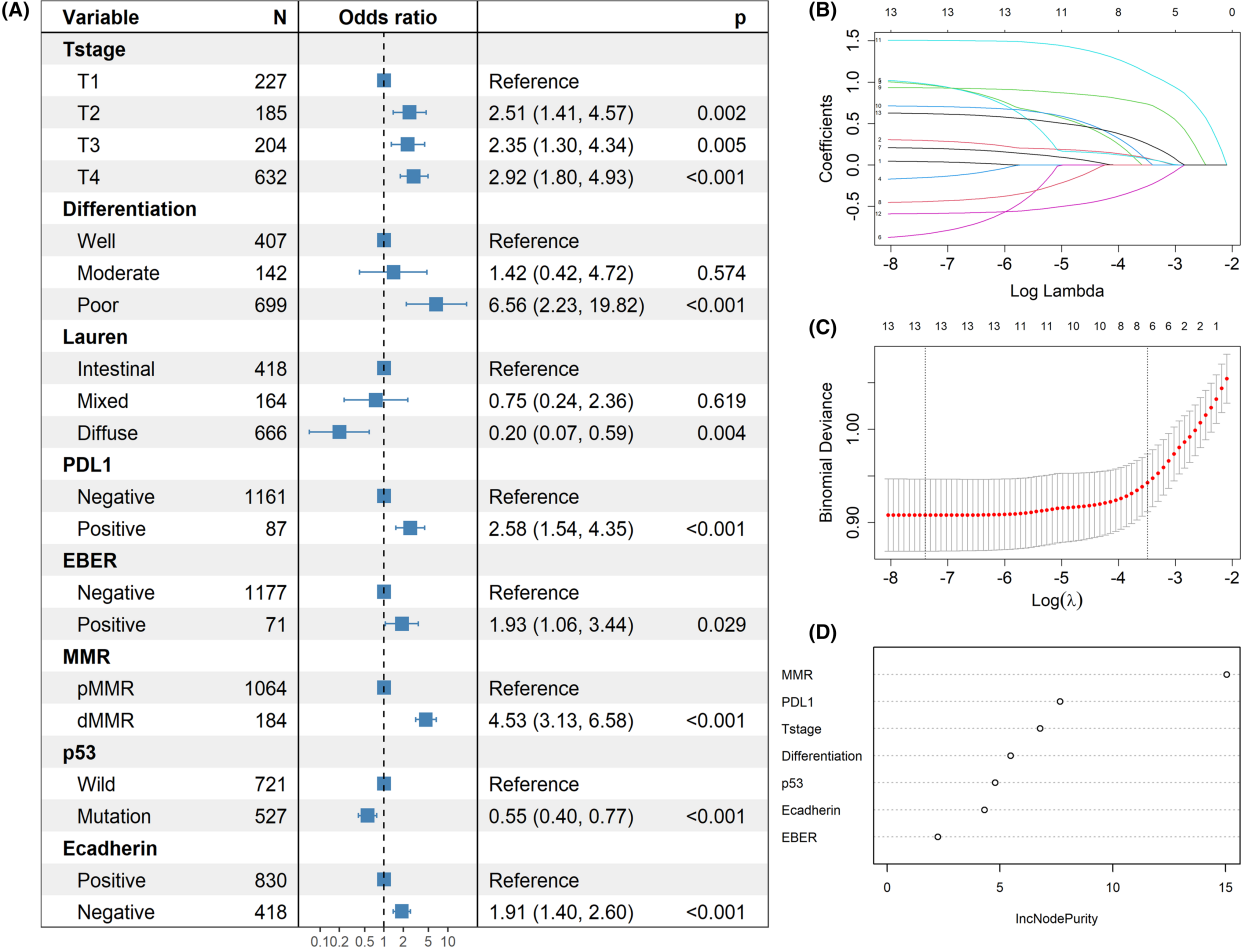
Correlation analysis of ARID1A. (A) Variables screened by multivariate logistic regression analysis. (B) and (C) Variables filtered out by the minimalist model of LASSO regression. (D) Variables ranked by importance using random forest. dMMR, mismatch repair deficient; EBER, EBV‐encoded RNA; MMR: mismatch repair; PD‐L1, programmed cell death ligand‐1; pMMR, mismatch repair proficient.

We performed multivariate logistic regression analysis (Figure 4A) and LASSO regression analysis (Figure 4B,C) for the above 13 variables, and screened out seven independent correlated variables, which were ranked using random forest according to the importance as follows: MMR, PD‐L1, T‐stage, differentiation status, p53, E‐cadherin, and EBER (Figure 4D). Details of variables filtered out by LASSO regression and the ranking of the variables by random forest are presented in Table S3.

### 3.3 Survival and prognostic analysis

**FIGURE 5:**
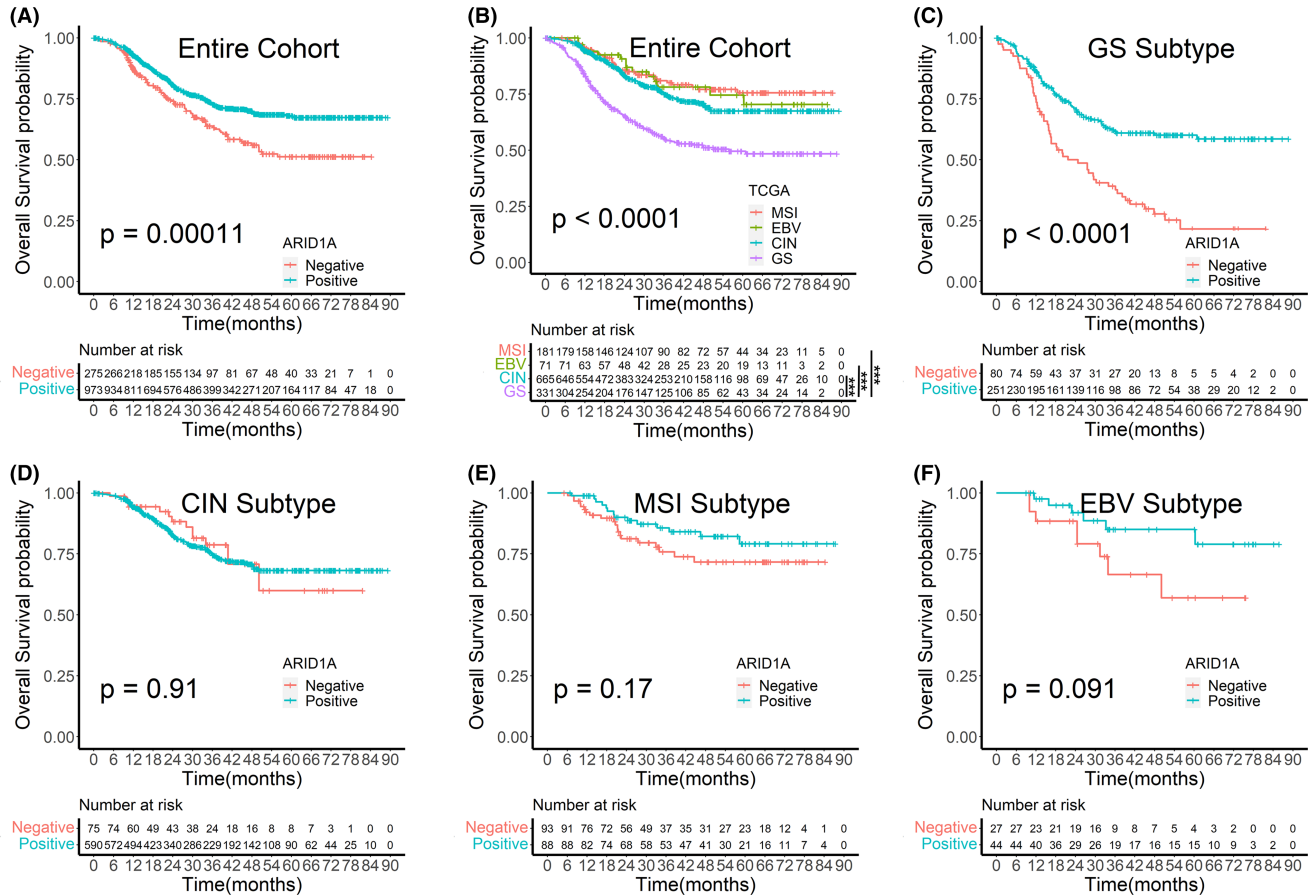
Survival analysis plots. (A) Survival analysis by ARID1A expression in the entire cohort. (B) Survival analysis by TCGA subtypes in the entire cohort. (C)–(F) Survival analysis by ARID1A expression in TCGA subtypes. CIN, chromosomal instability; EBV, Epstein–Barr virus; GS, genomically stable; MSI, microsatellite instability. ***: *p* < 0.001.

Univariate log‐rank survival analysis revealed that the ARID1A negative group showed a worse survival than the ARID1A positive group (*p* = 0.0001, Figure 5A). The GS subgroup exhibited a significantly worse survival than the other three subtypes (*p* < 0.0001, Figure 5B), and statistical results of pairwise comparisons between subgroups were shown in Table S4. We also performed survival analyses of ARID1A expression in each TCGA subtype and found that ARID1A negative group showed worse survival only in the GS subgroup (*p* < 0.0001, Figure 5C), whereas ARID1A negative was not associated with worse survival in the other three subtypes (Figure 5D–F).

**FIGURE 6:**
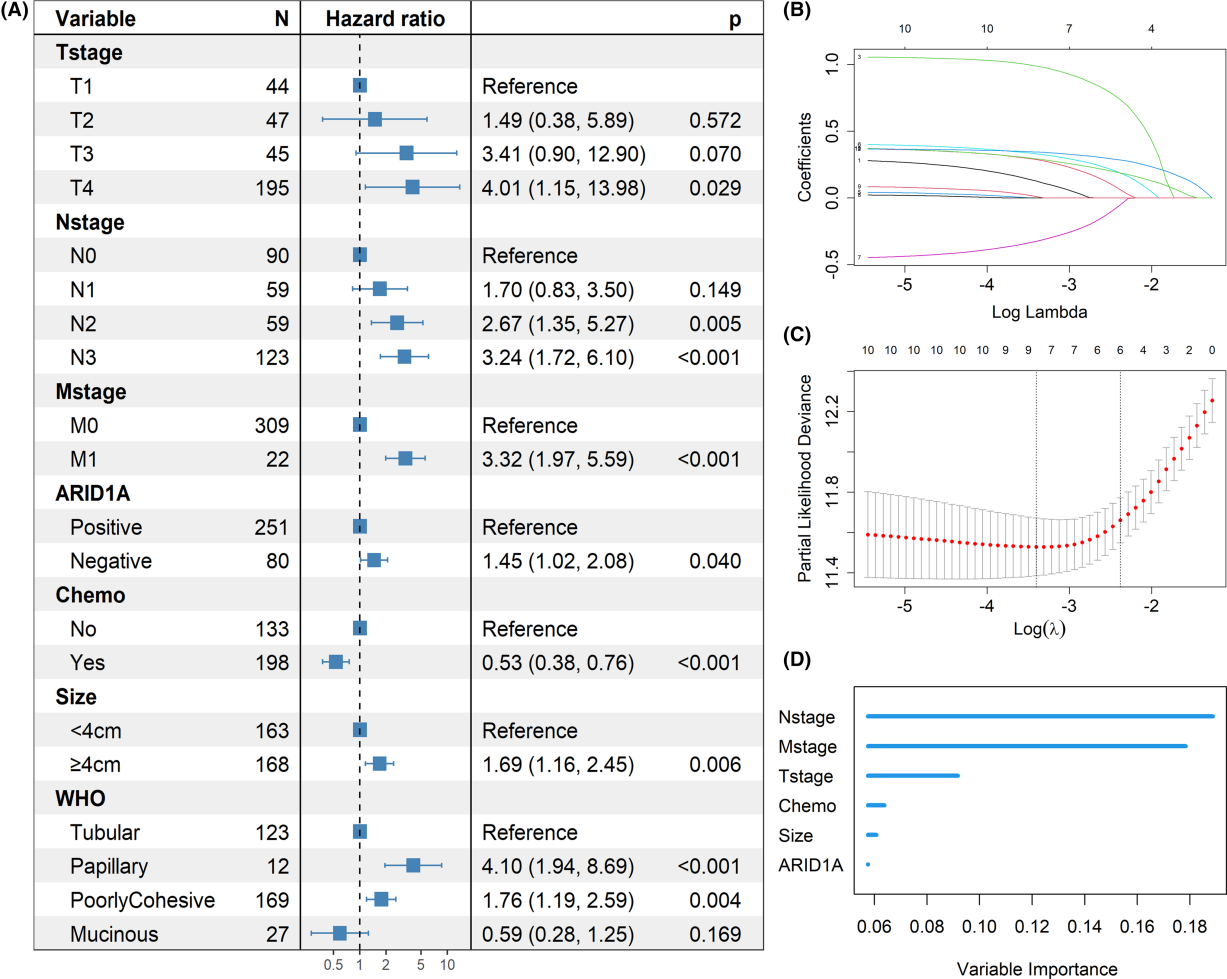
Prognostic analysis in the GS subtype. (A) Independent prognostic variables screened by univariate and multivariate Cox regression analysis. (B) and (C) Variables filtered out by the minimalist model of LASSO regression. (D) Variables ranked by importance using random survival forest. Chemo, chemotherapy.

We carried out univariate and multivariate Cox regression analyses in the entire cohort and found that ARID1A negative expression was an adverse factor for poor prognosis in the univariate Cox regression analysis, whereas this association disappeared after the multivariate Cox regression analysis (Table S5). We subsequently performed univariate Cox regression analysis in the GS subtype and screened out 11 variables associated with prognosis: age, size, VI, PNI, ARID1A, chemotherapy, WHO histological classification, differentiation status, T stage, N stage, and M stage (Table 2). Next, we performed multivariate Cox regression analysis and LASSO regression analysis for the above 11 variables and screened out six independent prognostic variables, which were ranked using a random survival forest according to the importance as follows: N stage, M stage, T stage, chemotherapy, size, and ARID1A (Figure 6D). The information of variables filtered out by LASSO regression and the ranking of the variables by random survival forest were presented in Table S6.

**TABLE 2:** Univariate and multivariate Cox regression analysis for overall survival in GS subtype.

Variables	Univariate Cox	*p‐*Value	Multivariate Cox	*p‐*Value
	HR (95% CI)		HR (95% CI)	
Sex (ref = female)				
Male	1.12 (0.75–1.66)	0.579		
Age (ref = “<60”)				
≥60	1.67 (1.11–2.50)	**0.013**	1.48(0.96–2.28)	0.076
Size (ref = “<4 cm”)				
≥4 cm	2.52 (1.78–3.56)	**<0.001**	1.64(1.12–2.39)	**0.01**
Site (ref = antrum)				
Body	1.15 (0.79–1.67)	0.460		
Cardia	1.45 (0.86–2.45)	0.163		
Differentiation (ref = well)				
Moderate	1.17 (0.60–2.27)	0.640	0.91(0.45–1.86)	0.797
Poor	1.57 (1.02–2.40)	**0.039**	1.25(0.69–2.28)	0.457
WHO (ref = tubular)				
Papillary	3.53 (1.71–7.26)	**0.001**	4.17(1.88–9.24)	**<0.001**
Poorly cohesive	1.65 (1.14–2.38)	**0.008**	1.61(0.97–2.69)	0.068
Mucinous	0.87 (0.42–1.78)	0.703	0.49(0.21–1.12)	0.09
Lauren (ref = intestinal)				
Mixed	0.88 (0.47–1.67)	0.706		
Diffuse	1.41 (0.93–2.13)	0.105		
T_stage (ref = T1)				
T2	2.62 (0.69–9.86)	0.156	1.48(0.37–5.84)	0.577
T3	10.90 (3.21–36.93)	**<0.001**	3.22(0.85–12.18)	0.084
T4	12.01 (3.81–37.82)	**<0.001**	3.92(1.12–13.75)	**0.033**
N_stage (ref = N0)				
N1	2.12 (1.05–4.26)	**0.035**	1.8(0.88–3.71)	0.109
N2	4.73 (2.52–8.87)	**<0.001**	2.5(1.25–5)	0.009
N3	6.84 (3.85–12.14)	**<0.001**	3.15(1.67–5.96)	**<0.001**
M_stage (ref = M0)				
M1	5.46 (3.3–9.02)	**<0.001**	3.65(2.13–6.24)	**<0.001**
VI (ref = no)				
Yes	1.91 (1.35–2.71)	**<0.001**	1.13(0.76–1.66)	0.555
PNI (ref = no)				
Yes	1.94 (1.39–2.71)	**<0.001**	0.99(0.69–1.42)	0.9385
Her‐2 (ref = negative)				
Positive	0.86 (0.42–1.75)	0.672		
Ki‐67 (ref = low)				
High	0.97 (0.63–1.49)	0.895		
PD‐L1 (ref = negative)				
Positive	1.49 (0.73–3.03)	0.277		
ARID1A (ref = positive)				
Negative	2.33 (1.66–3.26)	**<0.001**	1.44(1.01–2.08)	**0.04**
Chemo (ref = no)				
Yes	0.65 (0.46–0.90)	**0.010**	0.56(0.39–0.81)	**0.002**

Variables with *p* < 0.05 were shown in bold value.

Abbreviations: Chemo, chemotherapy; Her‐2, human epidermal growth factor receptor 2; PD‐L1, programmed cell death ligand‐1; PNI, perineural invasion; ref, reference; VI, vascular invasion.

### 3.4 Correlation of ARID1A with CD4 ^+^ and CD8 ^+^ T cells and PD‐L1 expression

After PSM and exclusion of spots without tumor tissue or detached from the slides, 107 ARID1A positive patients and 128 ARID1A negative patients were included in the mIF study. The distribution plot of propensity scores was shown in Figure S1, and comparisons of baseline characteristics after PSM were shown in Table S7.

**FIGURE 7:**
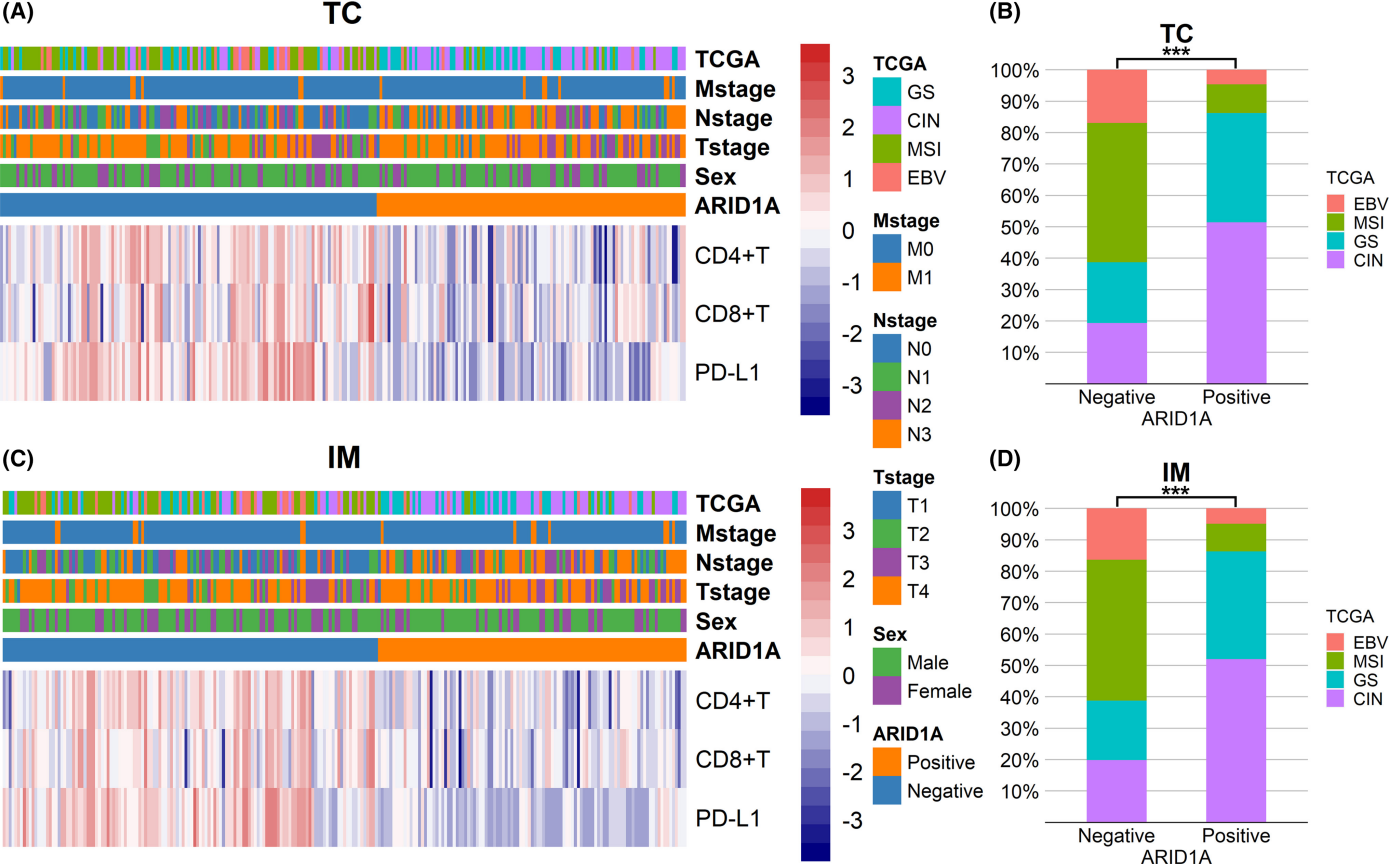
Heatmaps of CD4, CD8, and PDL1 expression and bar plots of TCGA subtypes distribution. (A) Heatmap of CD4, CD8, and PD‐L1 expression in tumor center. (B) Distribution of TCGA subtypes in ARID1A negative and positive groups in tumor center. (C) Heatmap of CD4, CD8, and PD‐L1 expression in invasive margin. (D) Distribution of TCGA subtypes in ARID1A negative and positive groups in invasive margin. CIN, chromosomal instability; EBV, Epstein–Barr virus; GS, genomically stable; IM, invasive margin; MSI, microsatellite instability; TC, tumor center; TCGA, The Cancer Genome Atlas. ***: *p* < 0.001.

**FIGURE 8:**
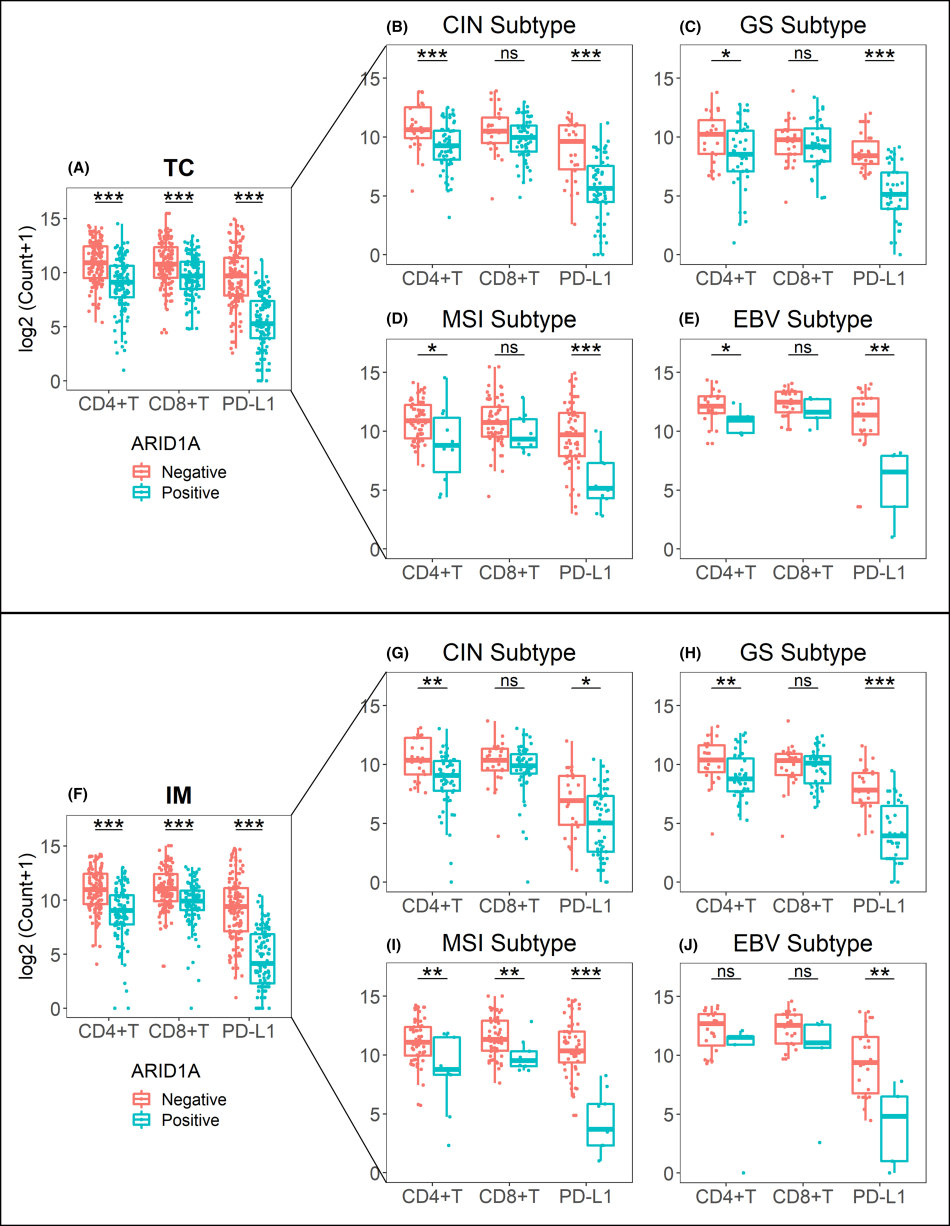
Impact of ARID1A status on the expression of CD4, CD8, and PD‐L1. (A) The entire mIF cohort in tumor center; (B)–(E) TCGA subtypes in tumor center; (F) the entire mIF cohort in invasive margin; (G)–(J) TCGA subtypes in invasive margin. CIN, chromosomal instability; EBV, Epstein–Barr virus; GS, genomically stable; IM, invasive margin; MSI, microsatellite instability; ns, no significance; TC, tumor center. *: *p* < 0.05; **: *p* < 0.01; ***: *p* < 0.001.

The heatmap showed that the expression of CD4, CD8, and PD‐L1 clustered into a high expression category (red predominant) and a low expression category (blue predominant); this means the ARID1A negative group had higher expression of CD4, CD8, and PD‐L1 than ARID1A positive group (Figure 7A,C). The barplots showed that the proportion of EBV and MSI subtypes was significantly higher in the ARID1A negative group than in the ARID1A positive group (Figure 7B,C). Detailed statistics results were shown in Table S8. We analyzed the correlation between ARID1A and immune infiltration after stratification according to TCGA classification. The boxplots showed that the ARID1A negative group had higher expression of CD4, CD8, and PD‐L1 than the ARID1A positive group in the entire cohort (Figure 8A,F). PD‐L1 expression was higher in the ARID1A negative group than in the ARID1A positive group in all TCGA subgroups. CD4 did not show differences in the EBV subtype of the IM (Figure 8J), while showed higher expression of the ARID1A negative group in the remaining subtypes. CD8 showed higher expression of the ARID1A negative group in the MSI subtype of the IM (Figure 8I), while did not show difference in the remaining subtypes.

**FIGURE 9:**
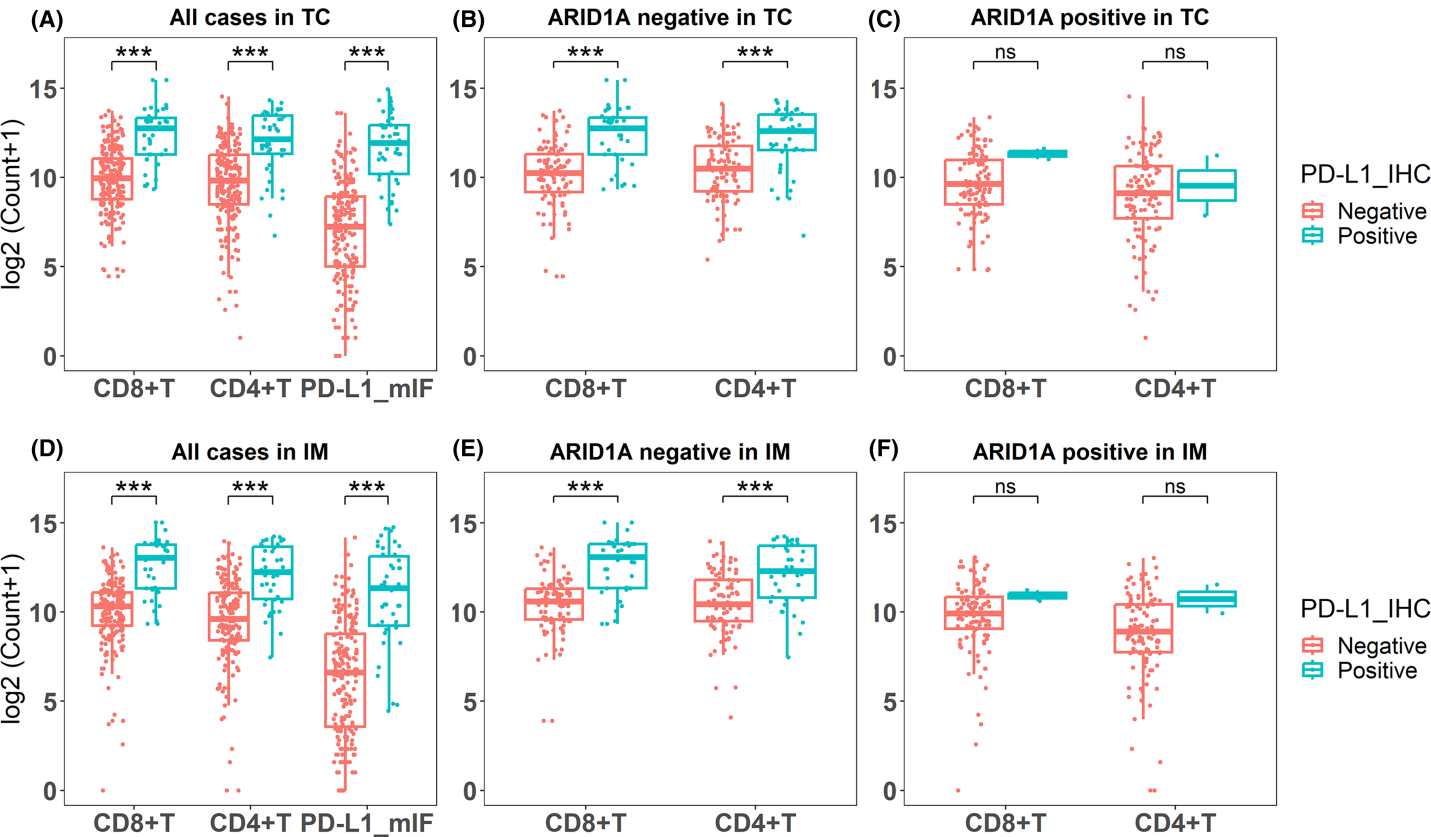
The correlation between PD‐L1 expression of IHC and immune infiltration. (A) and (D) The PD‐L1 expression in IHC was consistent with that in mIF; The expression of CD4/CD8 in the PD‐L1 positive group was significantly higher than that in PD‐L1 negative group for all cases. (B) and (E) When ARID1A was negative, the expression of CD4/CD8 in the PD‐L1 positive group was significantly higher than that in PD‐L1 negative group. (C) and (F) When ARID1A was positive, there was no difference in the expression of CD4/CD8 between PD‐L1 negative and PD‐L1 positive groups. IM, invasive margin; mIF, multiplex immunofluorescence; ns, no significance; TC, tumor center. *: *p* < 0.05; **: *p* < 0.01; ***: *p* < 0.001.

**FIGURE 10:**
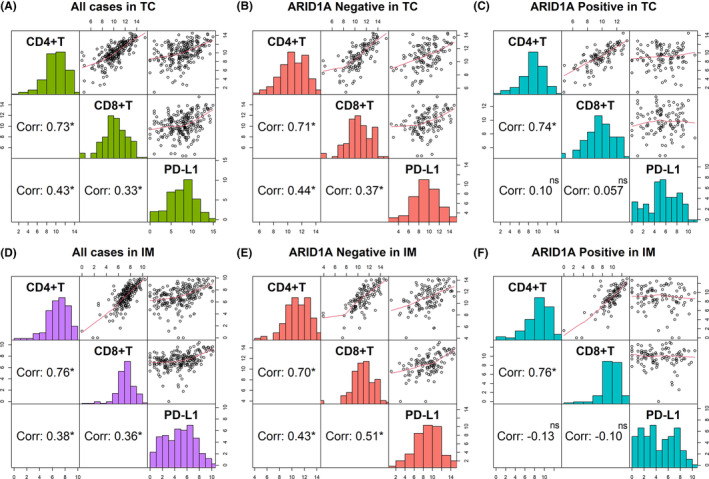
The correlation between PD‐L1 expression, CD4^+^, and CD8^+^ T cell infiltration in mIF. (A) and (D) PD‐L1, CD4, and CD8 were positively correlated in all cases; (B) and (E) PD‐L1, CD4, and CD8 were positively correlated in ARID1A negative cases; (C) and (F) CD4 and CD8 were positively correlated, while PD‐L1 was not associated with CD4 and CD8 in ARID1A positive cases. Corr, correlation coefficients; IM, invasive margin; ns, no significance; TC, tumor center; *: *p* < 0.05; **: *p* < 0.01; ***: *p* < 0.001.

**FIGURE 11:**
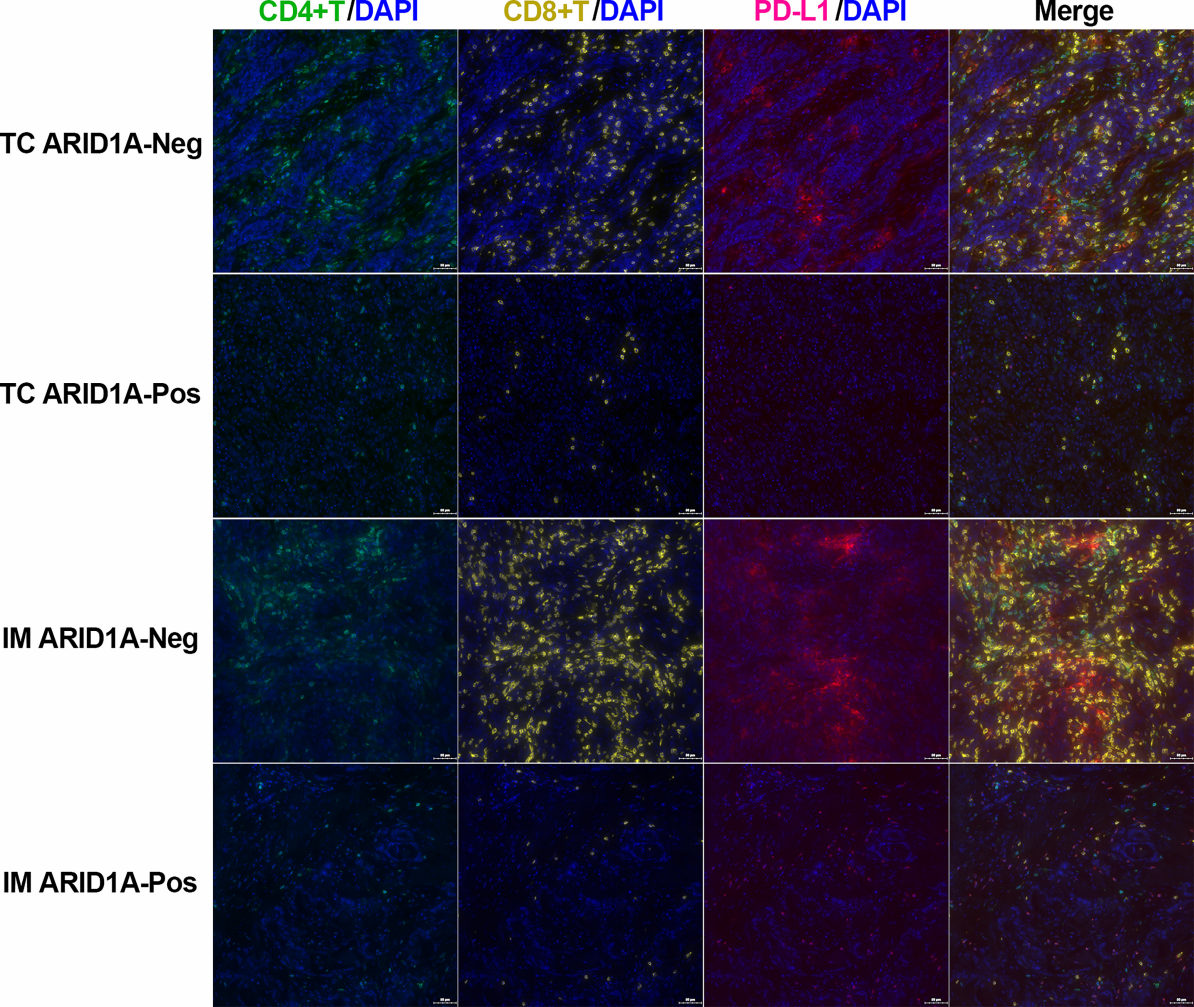
Representative mIF images according to the expression status of ARID1A in the tumor center and invasive margin. IM ARID1A‐Neg, invasive margin and ARID1A negative; IM ARID1A‐Pos, invasive margin and ARID1A positive; TC ARID1A‐Neg, tumor center and AIRD1A negative; TC ARID1A‐Pos, tumor center and AIRD1A positive.

We analyzed the correlation between PD‐L1 expression in IHC and immune infiltration. The results showed that the PD‐L1 expression in IHC was consistent with that in mIF (Figure 9A,D). When ARID1A was negative, the expression of CD4/CD8 in the PD‐L1 positive group was significantly higher than that in PD‐L1 negative group (Figure 9B,E), and when ARID1A was positive, this correlation disappeared (Figure 9C,F). Further, we analyzed the linear relationship among PD‐L1, CD4, and CD8 according to the data from mIF. We found a positive correlation between these three markers in ARID1A negative gastric carcinomas (Figure 10B,E), while PD‐L1 was not associated with CD4/ CD8 in ARID1A positive cases (Figure 10C,F). Typical images of mIF were shown in Figure 11.

## 4 DISCUSSION

*ARID1A* mutation has been observed in various tumors, particularly gynecological tumors, such as 57% of ovarian clear cell carcinomas^12^ and 25% of endometrial carcinomas.^13^ In gastric cancer, *ARID1A* has also been identified as a novel driver gene^14^ with a mutation frequency of 14%–24%.^2^, ^7^ The most common types of mutations were nonsense and frameshift mutations, leading to functional abnormalities of the ARID1A protein. Thus, negative expression of the ARID1A protein may reflect the mutation status of the *ARID1A* gene.^14^, ^15^ Many studies have confirmed that ARID1A expression is associated with multiple clinicopathological features of gastric cancer.^16^, ^17^ In this study, we screened out seven clinicopathological variables, among which MMR, EBER, and E‐cadherin were used for TCGA classification.

In this study, we found that the proportion of ARID1A negative expression varied significantly among different TCGA molecular subtypes, with higher ARID1A negative proportion in the MSI subtype and EBV subtype, illustrating the close correlation of ARID1A with MSI and EBV, which is consistent with previous TCGA findings.^2^ The coding region of the *ARID1A* gene contains many microsatellites.^14^ A study concerning exome sequencing of MSI colorectal cancers showed that the *ARID1A* gene exhibited more frequent short tandem repeats (STR) mutations, inferring MMR deficiency as the underlying cause of *ARID1A* mutations.^18^ However, a study of endometrial cancer found that ARID1A plays a role in epigenetic silencing (methylation) of the MLH1 gene, speculating that *ARID1A* mutations preceded MSI.^19^ Another proteomic study showed that ARID1A recruited MSH2 to chromatin during DNA replication and promoted MMR, whereas ARID1A inactivation led to MMR deficiency.^20^ Although the causal relationship between ARID1A and MSI is controversial, an increasing number of studies have shown that *ARID1A* was a causative gene for MSI rather than the target gene. A study confirmed that loss of ARID1A in EBV‐positive gastric cancer was partly regulated by EBV‐encoded miRNAs^21^; meanwhile, ARID1A loss increased the susceptibility of gastric epithelial cells to EBV infection and promoted gastric tumorigenesis.^22^ Several other variables related to ARID1A observed in this study were also confirmed in previous studies. For example, E‐cadherin expression was downregulated or absent when ARID1A was silenced,^23^ and the loss of E‐cadherin led to epithelial‐mesenchymal transition (EMT), further increasing tumor aggressiveness.^24^ ARID1A was inversely correlated with *TP53*, and simultaneous mutations in *ARID1A* and *TP53* were rarely observed in the same tumor.^14^, ^19^, ^25^ In addition, the loss of ARID1A caused worse differentiation and deeper tumor invasion in gastric cancer.^16^, ^17^

Loss of ARID1A resulted in higher T stage, worse differentiation, and E‐cadherin deficiency in this study, which are well‐known factors contributing to poor prognosis in gastric cancer. Interestingly, loss of ARID1A is closely associated with MSI and EBV, which can lead to immune infiltration, activate the immune system to kill tumor cells, and appear to be a favorable prognostic factor in gastric cancer. Although some studies of gastric cancer suggested that alterations in ARID1A caused better survival,^14^ more studies supported that loss of ARID1A was an adverse prognostic factor.^16^ Our present study showed that in the GS subtype, ARID1A negative expression resulted in worse OS and was an independent prognostic factor, whereas, in the other subtypes, ARID1A loss did not correlate with OS. This illustrated the complexity of the relationship between ARID1A and prognosis, which might be related to molecular background, treatment context, or potential confounders. In a study of liver cancer,^26^ ARID1A was also found to have context‐dependent tumor suppressor and oncogenic roles. Histologically, the GS subtype mainly shows the diffuse type in Lauren's classification, with E‐cadherin frequently absent and more prone to EMT, ultimately leading to a worse prognosis.^4^, ^27^ Our study also found poor survival in the GS subtype, and the ARID1A negative group in the GS subtype showed the worst survival, which may account for the poor survival of the entire cohort by ARID1A negative. The ARID1A negative group within the GS subtype represents a particular subtype with the worst prognosis and requires more in‐depth molecular‐level studies.

Some studies on gastric adenocarcinoma found SWI/SNF complex abnormalities to be an independent prognostic factor for OS in the GS subtype.^28^, ^29^ Interestingly, ARID1A negative expression exhibited worse survival in the EBV subtype,^29^ which was inconsistent with our present study, possibly due to different ARID1A classification methods or bias caused by too few patients in the subgroup. Large‐scale studies are needed to explore the complete molecular profile of gastric adenocarcinoma, confirm the prognostic significance of TCGA classification and ARID1A negative expression, and ultimately achieve risk stratification and more individualized patient management.

It has been well documented that the loss of ARID1A caused an increase in tumor‐infiltrating lymphocytes (TILs) and increased expression of PD‐L1.^30^ Our present study also reached the same conclusion. However, our study showed the proportions of the MSI subtype and EBV subtype were significantly higher in the ARID1A negative group than in the ARID1A positive group, and it has been confirmed that the MSI subtype and EBV subtype can lead to high PD‐L1 expression and high TILs.^31^ Therefore, to eliminate confounding factors, we performed subgroup analyses in TCGA subtypes and found that the expression of CD4 and PD‐L1 remained strongly associated with ARID1A expression in each subtype. In contrast, the expression of CD8 appeared to be independent of ARID1A status in each subtype. One study on ovarian cancer found that the relationship between ARID1A loss and CD8^+^ TILs was confounded by MMR status.^32^ In another study on ovarian cancer, ARID1A was found to directly inhibit the expression of CD274 (the gene encoding PD‐L1).^33^ More mechanistic studies related to immune regulation in gastric cancer are warranted. Our present study observed an inconsistent effect of ARID1A expression on the infiltration of CD4^+^ and CD8^+^ T cells in TCGA subtypes, implicating ARID1A involved in the immune infiltration through different mechanisms. Whether or to what extent the effects of ARID1A on TILs and PD‐L1 expression are influenced by confounding factors such as EBV and MMR is unclear, and more in‐depth investigations are still needed.

PD‐L1 expressed on tumor cells can inhibit the function of CD8^+^ T cells, leading to immune resistance and promoting tumor progression.^34^ Consistent with a previous study,^35^, ^36^ our study also found a high PD‐L1 expression in gastric cancer infiltrated with CD8^+^ T cells, suggesting that adaptive immune resistance is active and can be counteracted by inhibiting PD‐1/PD‐L1. Further hierarchical analysis found that PD‐L1 was highly expressed in gastric carcinomas with high CD8^+^ T infiltration only when ARID1A was negative. We speculated that the loss of ARID1A increased PD‐L1 expression by activating Akt signaling,^37^ further leading to immune resistance,^34^ which is also one of the reasons for the poor prognosis of ARID1A‐negative patients. Another plausible explanation for the poor prognosis of ARID1A‐negative patients is that ARID1A negativity induced the suppression of critical genes responsible for chemotherapy and radiotherapy sensitivity, leading to radioresistance or chemoresistance of cancer cells.^36^, ^38^

Numerous studies of targeted therapies in a synthetic lethality manner have been conducted based on *ARID1A* mutation,^39^ including poly polymerase, PI3K/AKT, Ataxia‐telangiectasia‐mutated‐and‐Rad3‐related kinase, enhancer of zeste homolog 2, and histone deacetylase 6, etc. Furthermore, due to the correlation between ARID1A and immune regulation, ARID1A may serve as a biomarker for immunotherapy.^40^

The limitations of this study are as follows. First, the TMA is an economical and highly efficient method for protein expression analysis, especially in large‐scale cohorts. However, there is a discrepancy between the results of protein expression obtained from TMA and the real results due to the heterogeneity of gastric cancer. Second, we used IHC and EBER‐ISH for TCGA classification instead of high‐throughput molecular techniques. However, protein expression may be insufficient to represent the complex molecular changes. Finally, we screened out a subset of patients for mIF using random sampling and PSM instead of the entire cohort, which may lead to biased statistical results.

## 5 CONCLUSIONS

In summary, we detected the IHC expression of ARID1A in gastric adenocarcinoma and explored the impact of ARID1A negative expression on prognosis and immune infiltration in combination with TCGA molecular classification. Negative expression of ARID1A occurred more frequently in the EBV and MSI subtypes and was an independent adverse prognostic factor in the GS subtype. The ARID1A negative group in the GS subtype may represent a particular population with the worst prognosis. The high CD8 expression caused by ARID1A negative was mainly due to MSI and EBV subtypes, whereas the high expression of PD‐L1 and CD4 resulted more from ARID1A itself. The increase of CD8^+^ T cells infiltration caused by ARID1A negativity was accompanied by the increase of PD‐L1 expression, which might induce adaptive immune resistance that can be resisted by inhibiting PD‐1/PD‐L1.

## AUTHOR CONTRIBUTIONS

**Zhenkun Zhang:** Conceptualization (supporting); data curation (equal); formal analysis (equal); investigation (equal); methodology (equal); validation (equal); writing – original draft (lead). **Qiujing Li:** Conceptualization (supporting); resources (equal); software (equal); validation (equal). **Shanshan Sun:** Data curation (equal); formal analysis (equal); investigation (equal); software (equal). **Jing Ye:** Conceptualization (supporting); resources (equal); validation (equal); writing – review and editing (supporting). **Zhe Li:** Data curation (equal); investigation (equal); writing – original draft (supporting). **Zhengguo Cui:** Methodology (equal); validation (equal); writing – review and editing (equal). **Qian Liu:** Resources (equal); software (equal). **Yujie Zhang:** Resources (equal); software (equal). **Sili Xiong:** Investigation (equal); software (equal). **Shukun Zhang:** Conceptualization (lead); data curation (equal); formal analysis (equal); methodology (equal); project administration (equal); supervision (equal); validation (equal); writing – review and editing (equal).

## FUNDING INFORMATION

None.

## CONFLICT OF INTEREST STATEMENT

The authors declare no conflicts of interest.

## ETHICS STATEMENT

This study was approved by the Ethics Review Board of the Weihai Municipal Hospital (permission code: 2021053). The Ethics Review Board considered that informed consent for this study was not required due to its retrospective design.

## Supporting information


Table S1.

Table S2.

Table S3.

Table S4.

Table S5.

Table S6.

Table S7.

Table S8.

Figure S1.

## Data Availability

The data are available on request from the corresponding author.
