# Supplementary material for: Prognostic and immune infiltration significance of ARID1A in TCGA molecular subtypes of gastric adenocarcinoma
Source: Cancer Med. 2023 Jun 27;12(16):16716–33. doi: 10.1002/cam4.6294 (PMC10501255; doi:10.1002/cam4.6294)
Supplement: Supplementary file 1 — Table S1. [file CAM4-12-16716-s005.docx]

Table S1. Assessment criteria for Her2, Ki-67 and PD-L1

| **Index** | **Location** | **Type** | | **Assessment Criteria** |
| --- | --- | --- | --- | --- |
| Her-2 (1) | Membrane | Negative | 0 | No reactivity or membranous reactivity in <10% of tumor cells |
|  |  |  | 1+ | Faint or barely perceptible membranous reactivity in ≥10% of tumor cells; cells are reactive only in part of their membrane |
|  |  | Equivocal | 2+ | Weak to moderate complete, basolateral or lateral membranous reactivity in ≥10% of tumor cells.  In situ hybridization was further performed, and cases with an average Her-2 copy number≥6.0 signals/cell were considered positive, or a ratio of HER2 signal to CEP17 signal of ≥ 2.0 is considered positive, and a ratio of HER2 signal to CEP17 signal<2.0 is considered negative. |
|  |  | Positive | 3+ | Strong complete, basolateral or lateral membranous reactivity in ≥10% of tumor cells |
| Ki-67 (2) | Nucleus | Low | 0 | Very low proliferation activity with proportion of Ki-67-positive cells <25% |
|  |  |  | 1+ | Low proliferation activity with proportion of Ki-67-positive cells 25–50% |
|  |  | High | 2+ | Moderate proliferation activity with proportion of Ki-67-positive cells 50–75% |
|  |  |  | 3+ | High proliferation activity with proportion of Ki-67-positive cells >75% |
| PD-L1 (3) | Membrane | Positive | | The presence of at least 1% of tumor cells with membrane staining, regardless of the intensity |
|  |  | Negative | | The tumor cell membrane did not show any staining |

**Reference**

1. Bartley AN, Washington MK, Colasacco C, Ventura CB, Ismaila N, Benson AB, 3rd, et al. HER2 Testing and Clinical Decision Making in Gastroesophageal Adenocarcinoma: Guideline From the College of American Pathologists, American Society for Clinical Pathology, and the American Society of Clinical Oncology [J]. *J Clin Oncol* (2017) 35(4):446-64. doi:10.1200/JCO.2016.69.4836

2. Wei Z, Huang L, Zhang X, Xu A. Expression and significance of Her2 and Ki-67 in gastric adenocarcinoma without distant metastasis: a cohort study [J]. *BMC Gastroenterol* (2020) 20(1):343. doi:10.1186/s12876-020-01484-9

3. Pereira MA, Ramos M, Faraj SF, Dias AR, Yagi OK, Zilberstein B, et al. Clinicopathological and prognostic features of Epstein-Barr virus infection, microsatellite instability, and PD-L1 expression in gastric cancer [J]. *J Surg Oncol* (2018) 117(5):829-39. doi:10.1002/jso.25022
