# Supplementary figures and images for: Prognostic and immune infiltration significance of ARID1A in TCGA molecular subtypes of gastric adenocarcinoma
Source: Cancer Med. 2023 Jun 27;12(16):16716–33. doi: 10.1002/cam4.6294 (PMC10501255; doi:10.1002/cam4.6294)

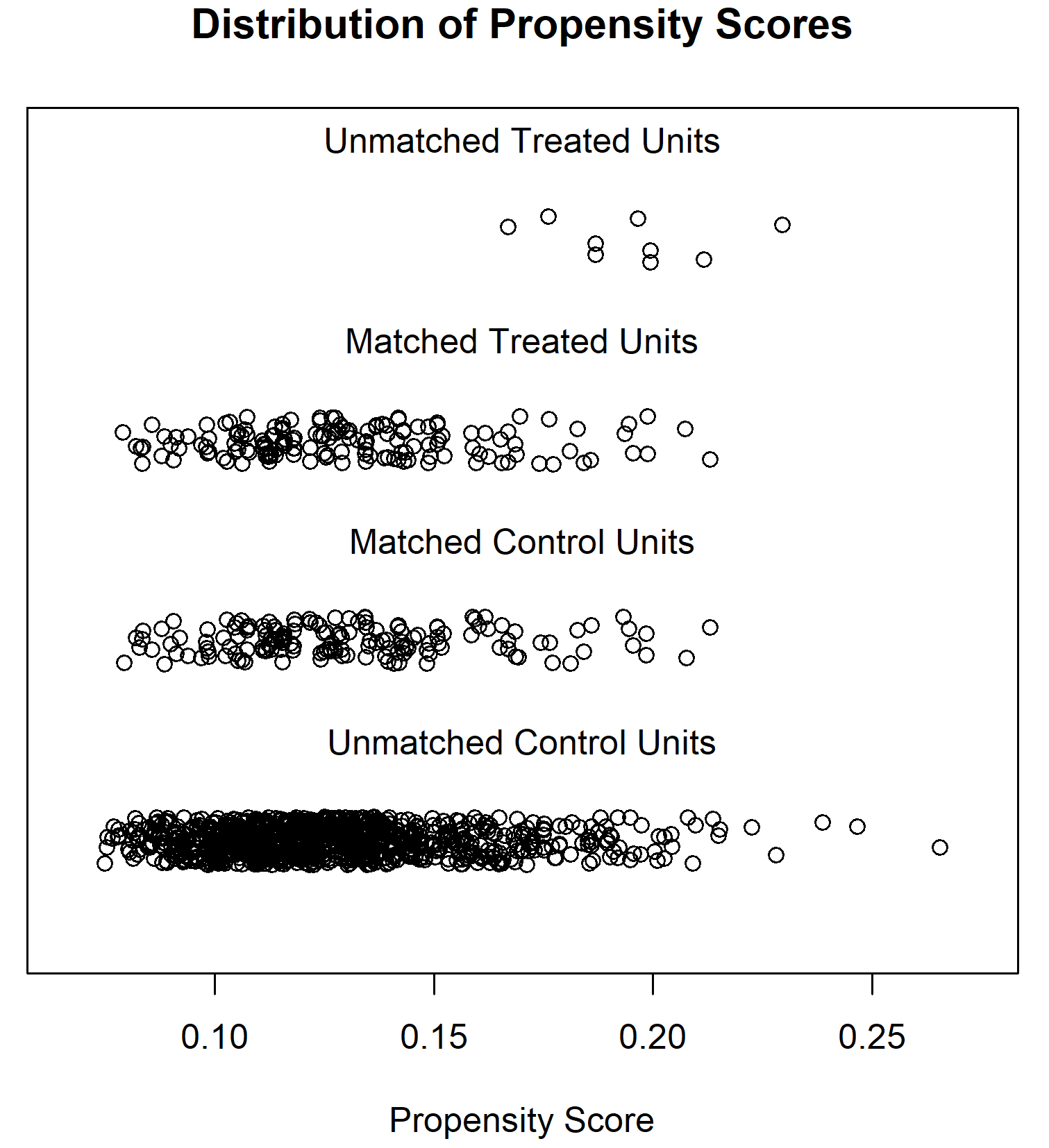

Supplement: Supplementary file 9 — Figure S1. [file CAM4-12-16716-s002.tiff]
